# Predicting in-hospital mortality among non-trauma patients based on vital sign changes between prehospital and in-hospital: An observational cohort study

**DOI:** 10.1371/journal.pone.0211580

**Published:** 2019-01-31

**Authors:** Yohei Kamikawa, Hiroyuki Hayashi

**Affiliations:** 1 Department of Emergency Medicine, University of Fukui Hospital, Fukui, Japan; 2 Department of Family Medicine, University of Fukui Hospital, Fukui, Japan; Monash University School of Public Health and Preventive Medicine, AUSTRALIA

## Abstract

**Objective:**

To prevent misjudgment of the severity of patients in the emergency department who initially seem non-severe but are in a critical state, methods that differ from the conventional viewpoint are needed. We aimed to determine whether vital sign changes between prehospital and in-hospital could predict in-hospital mortality among non-trauma patients.

**Methods:**

This observational cohort study was conducted in two tertiary care hospitals. Patients were included if they were transported by ambulance for non-trauma-related conditions but were excluded if they experienced prehospital cardiopulmonary arrest, were pregnant, were aged <15 years, had undergone inter-hospital transfer, or had complete missing data regarding prehospital or in-hospital vital signs. The main outcome was in-hospital mortality, and the study variables were changes in vital signs, pulse pressure, and/or shock index between the prehospital and in-hospital assessments. Logistic regression analyses were performed to obtain adjusted odds ratios for each variable. Receiver operating characteristic curve analyses were performed to identify cut-off values that produced a positive likelihood ratio of ≥2.

**Results:**

Among the 2,586 eligible patients, 170 died in the two hospitals. Significantly elevated risks of in-hospital mortality were associated with changes in the Glasgow Coma Scale (cut-off ≤–3), respiratory rate (no clinically significant cut-off), systolic blood pressure (cut-off ≥47 mmHg), pulse pressure (cut-off ≥55 mmHg), and shock index (cut-off ≥0.3).

**Conclusions:**

Non-trauma patients who exhibit changes in some vital signs between prehospital and in-hospital have an increased risk of in-hospital mortality. Therefore, it is useful to incorporate these changes in vital signs to improve triaging and predict the occurrence of in-hospital mortality.

## Introduction

There is limited to time to judge the severity or emergency of patients, especially in the crowded emergency department (ED). To prevent missing patients whose condition seems non-severe initially but is actually critical in a busy ED, measurement of vital signs in the ED is known to be useful for triaging patients who arrive via ambulance transportation. Prehospital vital signs are also informative for trauma patients in preparing immediate interventions.[1] This approach is advantageous in rural settings or in developing countries, as vital sign measurement is simpler, easier, and more rapid than blood testing.[2] Furthermore, a combination of vital signs can predict the risk of mortality, which has led to the development of various diagnostic tools.[2–5] Nevertheless, it is still difficult to accurately predict the risk of mortality based on a single vital sign, which typically cannot provide good specificity and sensitivity.[6] In addition, vital signs can vary considerably in emergent or special states, such as pregnancy, advanced age, intracranial disease, and chronic hypoxemia.[7–9] Thus, to not miss critical patients, development of a new warning tool that differs from the conventional viewpoint is needed.

One of the solutions to the problem is to consider changes in vital signs to assess the full episode of care from prehospital to in-hospital. Bruijns et al. revealed that mortality can be predicted using changes in the vital signs of trauma patients between prehospital and in-hospital.[10] However, although this has been hypothesized by many clinicians, no studies have been conducted to determine whether it is the case.

This study evaluated whether vital sign changes between prehospital and in-hospital could predict the risk of in-hospital mortality among non-trauma patients.

## Materials and methods

### Study design and setting

This observational cohort study was conducted in two urban tertiary hospitals that annually receive >2,500 and >4,000 cases via ambulance transportation. The population of the area for which these hospitals are responsible is about 777,000 people. The emergency medical services system is controlled by the local fire departments, and it provides emergency responses for calls. The emergency medical services system is an official agency composed of firefighter emergency medical technicians (EMTs) providing initial care and transport. EMTs are professionals qualified through national examinations. They are able to perform crystalloid resuscitation, intubation, blood sugar measurement, glucose infusion, and intramuscular or intravascular adrenalin injection depending on the circumstances. Emergency physicians maintain their high quality standards with on the job instructions and off the job feedback and discussions.

Written informed consent was exempted because of the retrospective observational nature of the study, and it was conducted using the opt-out method on the websites of these hospitals. All data were fully anonymized before we accessed them. This study’s protocol was approved by the research ethics committee of the University of Fukui Hospital (20160131) and the Fukui Prefectural Hospital (16–60). Patients were considered eligible if they were transported via ambulance for non-trauma-related conditions between July 2015 and June 2016. Patients were excluded if they experienced prehospital cardiopulmonary arrest, were pregnant, were <15 years old, had undergone inter-hospital transfer, or had complete missing data (e.g. no data were recorded at all) regarding prehospital or in-hospital vital signs.

### Study protocol

Prehospital and in-hospital data were collected regarding age, sex, transport time, oxygen use, diagnosis of chronic respiratory disease (chronic obstructive pulmonary disease, chronic bronchitis, asthma, and lung tumor) or intracranial disease (stroke, encephalitis, encephalopathy, seizure, and brain tumor), in-hospital mortality, and vital signs, which included body temperature (BT), heart rate (HR), systolic blood pressure (SBP), diastolic blood pressure (DBP), respiratory rate (RR), percutaneous arterial oxygen saturation (SpO_2_), Glasgow Coma Scale (GCS), pulse pressure (PP), and shock index (SI). The PP was calculated as the difference between SBP and DBP (PP = SBP–DBP) and the SI was calculated as the ratio of HR to SBP (SI = HR / SBP).[2] The emergency medical services were asked to measure single prehospital vital signs just before departure from the scene, then to record them manually. In-hospital single vital signs were measured by nurses or doctors immediately after the patient’s arrival, and the data were then fed into electronic medical records. The Bed Side Monitor BSM-3562 (NIHON KOHDEN, Tokyo, Japan) and the Bed Side Monitor PVM-2703 (NIHON KOHDEN, Tokyo, Japan) were used for measuring prehospital and in-hospital vital signs, respectively. These data were extracted from the patients’ electronic medical records and emergency service records. Missing data were replaced using the multiple imputation method.[11,12]

The main outcome was in-hospital mortality. Patients discharged from the ED were considered survivors. The study variables were calculated as changes between the prehospital and in-hospital values for each vital sign (e.g., ΔBT = in-hospital BT–prehospital BT). The absolute values of these changes (e.g., |ΔBT|) were also included based on the probability of a U-shaped relationship.[13] The secondary outcomes were cut-off values for each relevant variable that would produce a positive likelihood ratio (+LR) of ≥2 or a point closest to the (0,1) point in the receiver operating characteristic (ROC) curve.[10,14] The area under the ROC curve and the cut-off values were calculated based on significant variables that affected the main outcome and could provide clinically meaningful information.[14] The cut-off values were rounded to the nearest integer or to no more than one significant figure, especially for SI.

### Data analysis

Categorical variables were reported as the number and percentage, while continuous variables were reported as the median and interquartile range. The primary outcome (risk of in-hospital mortality compared with survivors for each change in vital sign) was evaluated using logistic regression analysis that was adjusted for age, sex, transport time, oxygen use, and in-hospital vital signs.[15] In-hospital vital signs and oxygen use were chosen for adjustment because these are variables of the National Early Warning Score, which is known as a reliable mortality predicting score using single vital signs.[5] By adjusting for these variables, this model aims to elucidate the predictability of vital sign changes independent of single vital signs. The variance inflation factor (VIF) of each change of vital sign was also calculated to verify the absence of multicollinearity.

In addition to the adjustment for the variables described above, we adjusted for chronic respiratory disease and intracranial disease in the logistic regression analysis to perform sensitivity analysis; in the sensitivity analysis we evaluated the effect of diseases whose vital signs present with different dynamics compared with other diseases.[6,9,16] We also used a generalized estimating equation (GEE) without using the imputation method to perform another sensitivity analysis to assess the effects of missing data. All analyses were performed using R software, version 3.4.1 (The R Foundation, Vienna, Austria).

## Results

### Characteristics of study participants

**Fig 1:**
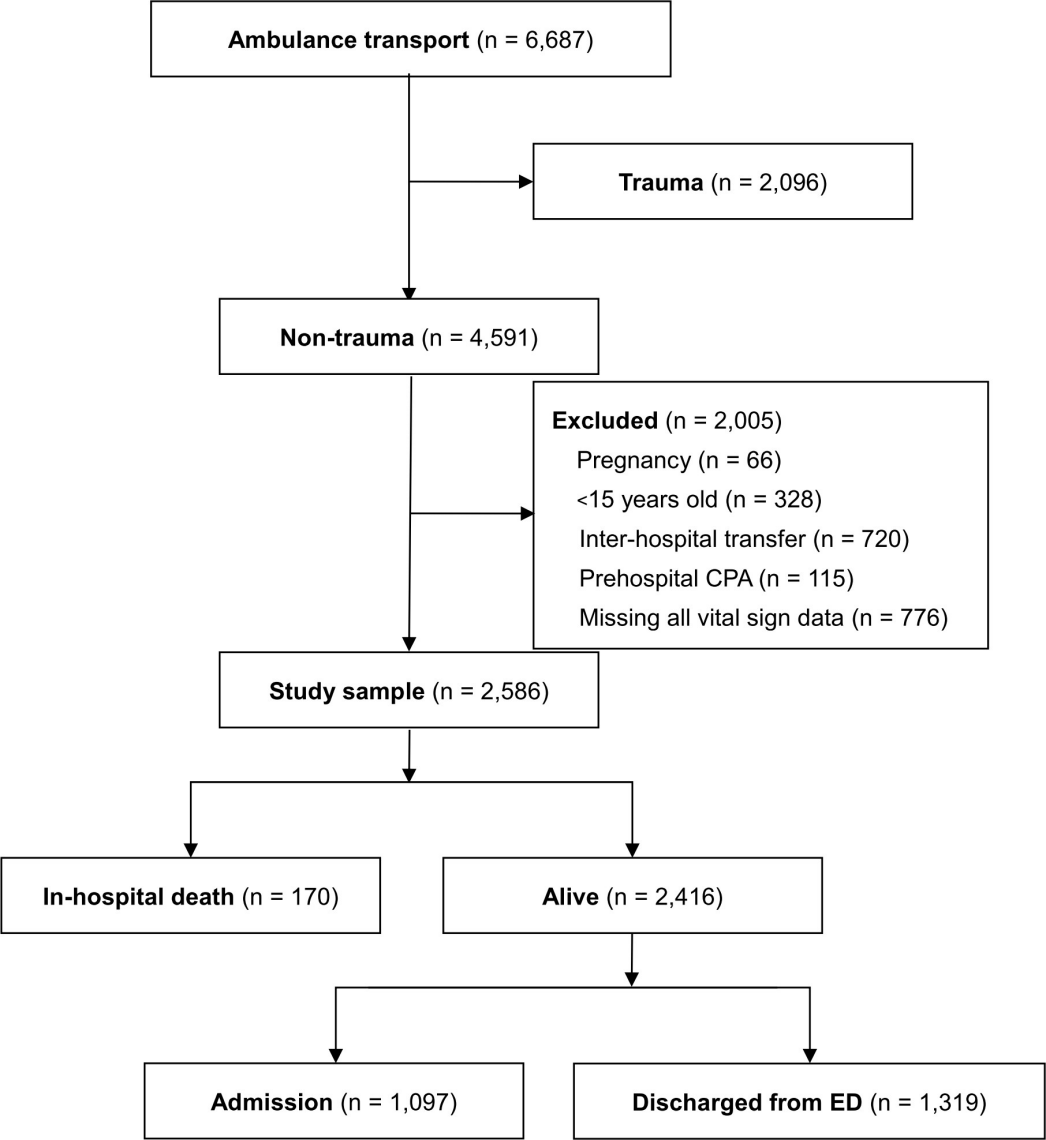
Study flow chart. CPA: cardiopulmonary arrest, ED: emergency department.

During the study period, 6,687 patients were transported to the two hospitals by ambulance, including 4,591 non-trauma patients. However, the exclusions included 115 patients with prehospital cardiopulmonary arrest, 66 pregnant patients, 328 patients who were <15 years old, 720 patients who required involved inter-hospital transfer, and 776 patients who had complete missing vital sign data at the prehospital or in-hospital evaluations. Thus, data from 2,586 patients were evaluated, including 170 patients who died in the hospitals, while the others improved and were discharged from hospital by September 2016. Among 2,416 surviving patients, 1,319 were discharged from the ED without admission (Fig 1). The patients’ characteristics and vital signs are summarized in Table 1 and Table 2. The patients had a relatively old median age (72 years) and a short median transport time (12 min). There were no significant sex-based differences. The overall missing values rate was 12.9% and this was addressed using multiple imputation, although RR and BT in particular had many missing values (around 50% and 30%, respectively). Vital signs regarding outcomes are summarized in S1 Table.

**Table 1:** Patient characteristics.

	Median (IQR)	Data missing (%)
Age, years	72 (53–83)	0 (0)
Transport time, min	12 (7–28)	413 (16.0)
	N (%)	Data missing (%)
Sex		
Female	1,283 (49.6)	0 (0)
Male	1,304 (50.4)	0 (0)
Oxygen demand	597 (23.1)	0 (0)
Chronic respiratory disease	80 (3.1)	0 (0)
Intracranial disease	331 (12.8)	0 (0)

IQR: interquartile range.

**Table 2:** Vital signs.

	Prehospital		Emergency department	
	Median (IQR)	Data missing (%)	Median (IQR)	Data missing (%)
BT	36.5 (36.0–37.1)	913 (35.3)	36.6 (36.2–37.0)	583 (22.5)
HR	85 (72–100)	129 (5.0)	84 (71–99)	168 (6.5)
SBP	139 (118–164)	90 (3.5)	139 (119–163)	115 (4.4)
DBP	79 (65–93)	104 (4.0)	80 (68–93)	136 (5.3)
RR	20 (18–24)	1542 (59.6)	20 (16–24)	1251 (48.4)
SpO_2_	98 (95–99)	93 (3.6)	98 (96–99)	265 (10.2)
GCS	15 (15–15)	229 (8.8)	15 (14–15)	649 (25.1)

IQR: interquartile range, BT: body temperature, HR: heart rate, SBP: systolic blood pressure, DBP: diastolic blood pressure, RR: respiratory rate, SpO2: percutaneous arterial oxygen saturation, GCS: Glasgow Coma Scale.

The results of the logistic regression analyses are shown in Table 3. All VIFs of each study variable were under 2.50, which implies independence and absence of multicollinearity. Significant risks of in-hospital mortality were associated with ΔRR, ΔGCS, |ΔSBP|, |ΔPP|, and |ΔSI|. The results of the ROC curve analyses are shown in Table 4. The cut-off points to provide a +LR of ≥2 were ≤–3 for ΔGCS, ≥47 mmHg for |ΔSBP|, ≥55 mmHg for |ΔPP|, and ≥0.3 for |ΔSI|. There was no clinically significant cut-off point for ΔRR.

**Table 3:** Logistic regression analyses of vital sign changes for predicting in-hospital mortality.

	VIF	Unadjusted OR (95% CI)	Adjusted OR (95% CI)
ΔBT	1.1564	1.1836 (0.8538–1.6406)	1.2372 (0.8838–1.7319)
|ΔBT|	1.0388	1.2162 (0.8891–1.6636)	1.1491 (0.8432–1.5660)
ΔHR	1.1191	1.0034 (0.9919–1.0150)	1.0085 (0.9971–1.0201)
|ΔHR|	1.0418	0.9995 (0.9860–1.0132)	0.9949 (0.9816–1.0085)
ΔSBP	1.1495	0.9978 (0.9912–1.0044)	0.9964 (0.9893–1.0036)
**|ΔSBP|**	1.0268	1.0086 (1.0022–1.0152) *	**1.0067 (1.0002–1.0135)** *
ΔDBP	1.1080	0.9893 (0.9801–0.9987) *	0.9910 (0.9806–1.0015)
|ΔDBP|	1.0470	1.0093 (0.9991–1.0197)	1.0058 (0.9954–1.0163)
**ΔRR**	1.5017	1.0550 (0.9973–1.1161)	**1.0683 (1.0082–1.1320)** *
|ΔRR|	1.0599	1.0179 (0.9667–1.0719)	1.0113 (0.9591–1.0664)
ΔSpO_2_	1.2315	1.0362 (1.0055–1.0677) *	0.9930 (0.9556–1.0318)
|ΔSpO_2_|	1.1296	1.0336 (1.0006–1.0677) *	0.9866 (0.9466–1.0284)
**ΔGCS**	1.3641	0.8525 (0.7892–0.9208) *	**0.8467 (0.7868–0.9113)** *
|ΔGCS|	1.3767	1.1380 (1.0586–1.2233) *	1.0732 (0.9902–1.1632)
ΔPP	1.2260	1.0045 (0.9964–1.0127)	1.0011 (0.9918–1.0104)
**|ΔPP|**	1.0255	1.0108 (1.0020–1.0197) *	**1.0102 (1.0012–1.0192)** *
ΔSI	1.1831	1.7189 (0.6466–4.5696)	2.0895 (0.8694–5.0221)
**|ΔSI|**	1.1279	4.8336 (2.1082–11.0819) *	**2.8442 (1.1581–6.9852)** *

VIF: variance inflation factor, OR: odds ratio, CI: confidence interval, BT: body temperature, HR: heart rate, SBP: systolic blood pressure, DBP: diastolic blood pressure, RR: respiratory rate, SpO2: percutaneous arterial oxygen saturation, GCS: Glasgow Coma Scale, PP: pulse pressure, SI: shock index.

|  | indicates the absolute value.

* Significant results based on the 95% CI values.

Bold font is used to indicate independent predictors after adjustment for age, sex, transport time, oxygen use, and the in-hospital values for BT, HR, SBP, DBP, RR, SpO2, and GCS.

**Table 4:** Receiver operating characteristic curve analyses of vital sign changes.

	AUROC (95% CI)	Minimum 0/1 distance				+LR of ≥2			
		Cut-off	+LR (95% CI)	Sensitivity (95% CI)	Specificity (95% CI)	Cut-off	+LR (95% CI)	Sensitivity (95% CI)	Specificity (95% CI)
**ΔGCS**	0.5400 (0.5300–0.5500)	≤0	1.3465 (1.0812–1.6768) *	0.34 (0.28–0.42)	0.75 (0.73–0.76)	**≤–3**	2.3970 (1.4319–4.0125) *	0.09 (0.05–0.14)	0.96 (0.95–0.97)
**|ΔSBP|**	0.5300 (0.5200–0.5410)	≥7	1.0953 (0.9415–1.2742)	0.52 (0.44–0.59)	0.53 (0.51–0.55)	**≥47**	2.0022 (1.3511–2.9671) *	0.14 (0.10–0.20)	0.93 (0.92–0.94)
**|ΔPP|**	0.5348 (0.5240–0.5450)	≥11	1.1657 (0.9701–1.4009)	0.42 (0.35–0.50)	0.64 (0.62–0.66)	**≥55**	2.1051 (1.1130–3.9813) *	0.06 (0.03–0.11)	0.97 (0.96–0.98)
**|ΔSI|**	0.5422 (0.5320–0.5530)	≥0.05	1.1857 (1.0222–1.3753) *	0.53 (0.46–0.61)	0.55 (0.53–0.57)	**≥0.3**	2.1718 (1.4787–3.1900) *	0.15 (0.10–0.21)	0.93 (0.92–0.94)
ΔRR	0.5733 (0.5630–0.5830)	≥0	1.1217 (0.9741–1.2916)	0.55 (0.47–0.63)	0.51 (0.49–0.53)	≥14	3.1582 (0.6878–14.501)	0.01 (0.00–0.04)	1.00 (0.99–1.00)

AUROC: area under the receiver operating characteristic curve, CI: confidence interval, +LR: positive likelihood ratio, SBP: systolic blood pressure, RR: respiratory rate, GCS: Glasgow Coma Scale, PP: pulse pressure, SI: shock index.

* Significant results based on the 95% CI values.

| | indicates the absolute value.

Bold font is used to indicate significant cut-off values that provide a positive likelihood ratio of two or more.

In the sensitivity analysis adjusted for chronic respiratory disease and intracranial disease, significant risks were observed for ΔRR, ΔGCS, |ΔPP|, and |ΔSI| (S2 Table); therefore, their robustness regarding diseases was confirmed. In the GEE-based sensitivity analyses, significant risks were observed for |ΔSBP|, |ΔPP|, and |ΔBT| (S3 Table). Robustness regarding missing data was confirmed for |ΔSBP| and |ΔPP| in the analyses.

## Discussion

To identify critical patients who seem non-severe in the ED, vital sign measurements are indispensable during the triage process, although using a single evaluation is associated with limitations. In this context, changes in vital signs are known to predict mortality among trauma patients, although we are not aware of any previous reports regarding non-trauma patients. Thus, the present observational cohort study evaluated the predictive ability of changing vital signs of non-trauma patients between prehospital and in-hospital and revealed that some of these factors could predict in-hospital mortality. Furthermore, clinically meaningful cut-off points were identified for ΔGCS, |ΔSBP|, |ΔPP|, and |ΔSI|. The cut-off point of 0.3 for |ΔSI| is very useful in clinical settings because it conveys a slight change but could be critical (e.g., changing from HR 65 and SBP 130 to HR 88 and SBP 110). In addition, although attention has only been paid to dropping SBP or rising SI, we found that the reverse trends were no less important.

It is generally considered that vital signs of elderly people hardly worsen even when their condition is critical,[17] and that the measurement of single vital signs is not useful for them. In contrast, some of the changes in vital signs showed significance independent of age. Therefore, they should be useful for improving current triaging protocols. Moreover, evaluating the vital sign changes will be useful in the future as society ages. Further, except for |ΔSBP|, our sensitivity analyses were adjusted for two kinds of diseases and revealed the robustness of ΔRR, ΔGCS, |ΔPP|, and |ΔSI|. Regarding the |ΔSBP|, it is possible that the characteristic elevation of blood pressure against intracranial pressure, especially in intracranial disease, was a confounding factor; therefore, it could not maintain its robustness. Another GEE-based sensitivity analysis revealed consistent results for |ΔSBP| and |ΔPP|, in which relatively few values were missing. It is conceivable that the inconsistency, except for |ΔSBP| or |ΔPP|, was generated from a high amount of missing data. It is not possible to conclude that the values, except for |ΔSBP| or |ΔPP|, are not significant because the GEE is only valid when the data are missing completely at random[18]; however, the effect of the missing data should be considered.

To the best of our knowledge, this is the first study to investigate the ability of changing vital signs to predict mortality among transported non-trauma patients. This is because most related studies evaluated trauma patients or non-trauma patients who were hospitalized rather than transported.[19–21] Furthermore, there was no standardized protocol for measuring the variables, and some studies had excluded all patients with missing data,[10,22] while others used a single imputation method without multiple imputation.[21] Moreover, various endpoints were used, including survival to 48 h,[10] the requirement for blood transfusion,[19,23] or a composite outcome of intensive care unit transfer, cardiac arrest, and death.[21] Our findings are generally in agreement with the findings of those studies, although it is difficult to compare the results based on those differences.

There are two general explanations for changes in vital signs, with one being directionality and the other being instability. The directionality explanation assumes that patients who are in a critical state have vital signs that increase or decrease directionally.[24] Related studies have indicated that the clinically meaningful cut-off points are a ΔRR of ≥8, ΔGCS of ≤–2, ΔSBP of ≤–26 to ≤–37 mmHg, and ΔSI of ≥0.1 to ≥0.3.[10,20,22] These values are smaller than those of our study, which is likely related to underestimation caused by the short observation time (transportation time) that makes it more difficult to detect significant changes.

The instability explanation assumes that the vital signs of patients in a critical state will exhibit bi-directional fluctuation.[23] For example, SI exhibits a U-shaped relationship with mortality among trauma patients.[13] Interestingly, another study revealed that changes in vital signs reduced the ability to detect critical illness in the wards, although the 24 h standard deviation of the change improved the predictive ability,[21] which suggests that a U-shaped relationship influenced the result. Another study evaluated massive bleeding and revealed that RR, SBP, and SI exhibited instability during the transport time, while PP, HR, and SpO_2_ did not exhibit instability.[23] That result is consistent with our findings that the absolute values of ΔSBP and ΔSI were significant predictive factors.

The present study had several limitations. It was conducted at two hospitals in a single region, and the findings may not be generalizable to other populations and regions. For example, the patients were relatively old (median age: 72 years) and had a short median transport time (12 min); however, these factors were adjusted for. As it can be difficult to detect changing vital signs during a short transport time, it is possible that our findings underestimate the predictive ability of vital sign changes. Next, follow-up for patients discharged from the ED could not be performed because data were fully anonymized before our analyses. This might have lead underestimate of predictability of vital sign changes. In contrast, there were many missing values for RR and BT, like in previous reports.[25,26] Besides, our sensitivity analyses unexpectedly revealed the necessity of multiple imputation even for SI. Though they were compensated for using multiple imputation, the possibility of selection bias still remains, especially in RR where the amount of missing data reached almost 50%. In addition, we were unable to control for all confounding factors based on the limitations of logistic regression analysis,[15] and it is possible that we underestimated the effects of factors that were not included (e.g., disease, medical history, and intubation or other treatments). Furthermore, we did not consider the physiological mechanisms that would explain the influence of changing vital signs on mortality. These issues should be addressed in future studies to determine how vital sign changes improve prediction of mortality and the extent of any improvement.

In conclusion, changes in some vital signs between prehospital and in-hospital can predict the risk of in-hospital mortality among non-trauma patients. Our analyses indicate that significant cut-off points (providing a +LR of ≥2) independent from single vital signs were observed for ΔGCS, |ΔSBP|, |ΔPP|, and |ΔSI|. Therefore, we recommend considering these factors in current triage protocols to improve the prediction of in-hospital mortality, especially in cases in which the level of emergency cannot be decided with single vital signs. Nevertheless, further studies are needed to examine how vital sign changes can improve this predictive ability and determine the magnitude of any improvement.

## Supporting information

S1 Table Vital signs regarding outcomes. (PDF)

S2 Table Sensitivity analyses adjusted for chronic respiratory disease and intracranial disease. (PDF)

S3 Table Sensitivity analyses using generalized estimating equations. (PDF)
