# Supplementary material for: Predicting in-hospital mortality among non-trauma patients based on vital sign changes between prehospital and in-hospital: An observational cohort study
Source: PLoS One. 2019 Jan 31;14(1):e0211580. doi: 10.1371/journal.pone.0211580 (PMC6355016; doi:10.1371/journal.pone.0211580)
Supplement: S1 Table — (PDF) [file pone.0211580.s001.pdf]

S1 Table. Vital signs regarding outcomes.

	Prehospital, Median (IQR)				Emergency department, Median (IQR)			
	In-hospital death	Admission	Discharged from ED	Total	In-hospital death	Admission	Discharged from ED	Total
BT	36.6 (36.2–37.4)	36.6 (36.1–37.2)	36.5 (36.1–37.0)	36.5 (36.0–37.1)	36.8 (36.4–37.5)	36.6 (36.2–37.2)	36.6 (36.3–36.9)	36.6 (36.2–37.0)
HR	91 (76–114)	87 (73–102)	83 (71–99)	85 (72–100)	90 (73–107)	85 (72–100)	82 (71–96)	84 (71–99)
SBP	137 (115–164)	142 (120–167)	138 (118–163)	139 (118–164)	135 (113–164)	141 (120–166)	139 (119–161)	139 (119–163)
DBP	78 (63–94)	79 (65–95)	78 (66–92)	79 (65–93)	76 (64–92)	81 (68–95)	80 (68–92)	80 (68–93)
RR	20 (18–24)	20 (18-24)	20 (18–24)	20 (18–24)	20 (18–24)	18 (16–23)	18 (16–22)	20 (16–24)
SpO_2	97 (93–99)	98 (95–99)	98 (96–99)	98 (95–99)	98 (95–99)	98 (95–99)	98 (96–99)	98 (96–99)
GCS	15 (15–15)	15 (15–15)	15 (15–15)	15 (15–15)	15 (14–15)	15 (15–15)	15 (15–15)	15 (14–15)

IQR: interquartile range, ED: Emergency Department, BT: body temperature, HR: heart rate, SBP: systolic blood pressure, DBP: diastolic blood pressure, RR: respiratory rate, SpO2: percutaneous arterial oxygen saturation, GCS: Glasgow Coma Scale.
