# Supplementary material for: Predicting in-hospital mortality among non-trauma patients based on vital sign changes between prehospital and in-hospital: An observational cohort study
Source: PLoS One. 2019 Jan 31;14(1):e0211580. doi: 10.1371/journal.pone.0211580 (PMC6355016; doi:10.1371/journal.pone.0211580)
Supplement: S2 Table — (PDF) [file pone.0211580.s002.pdf]

S2 Table. Sensitivity analyses adjusted for chronic respiratory disease and intracranial disease.

	Adjusted OR (95% CI)
ΔBT	1.2542 (0.8912–1.7650)
|ΔBT|	1.1545 (0.8442–1.5789)
ΔHR	1.0059 (0.9986–1.0217)
|ΔHR|	0.9931 (0.9795–1.0070)
ΔSBP	0.9961 (0.9889–1.0033)
|ΔSBP|	1.0065 (0.9998–1.0132)
ΔDBP	0.9905 (0.9801–1.0011)
|ΔDBP|	1.0053 (0.9948–1.0159)
**ΔRR**	**1.0672 (1.0062–1.1319)** *
|ΔRR|	1.0101 (0.9573–1.0658)
ΔSpO_2	0.9937 (0.9565–1.0324)
|ΔSpO_2|	0.9832 (0.9432–1.0280)
**ΔGCS**	**0.8539 (0.7918–0.9208)** *
|ΔGCS|	1.0648 (0.9815–1.1550)
ΔPP	1.0009 (0.9915–1.0103)
**|ΔPP|**	**1.0098 (1.0007–1.0190)** *
ΔSI	2.3143 (0.9585–5.5880)
**|ΔSI|**	**2.7798 (1.1201–6.8990)** *

OR: odds ratio, CI: confidence interval, BT: body temperature, HR: heart rate, SBP: systolic blood pressure, DBP: diastolic blood pressure, RR: respiratory rate, SpO_2: percutaneous arterial oxygen saturation, GCS: Glasgow Coma Scale, PP: pulse pressure, SI: shock index.

| | indicates the absolute value.

* Significant results based on the 95% CI values.

Bold font is used to indicate independent predictors after adjustment for age, sex, transport time, oxygen use, chronic respiratory disease, intracranial disease, and the in-hospital values for BT, HR, SBP, DBP, RR, SpO_2, and GCS.
