# Supplementary material for: Predicting in-hospital mortality among non-trauma patients based on vital sign changes between prehospital and in-hospital: An observational cohort study
Source: PLoS One. 2019 Jan 31;14(1):e0211580. doi: 10.1371/journal.pone.0211580 (PMC6355016; doi:10.1371/journal.pone.0211580)
Supplement: S3 Table — (PDF) [file pone.0211580.s003.pdf]

**S3 Table. Sensitivity analyses using generalized estimating equations.**

| | Adjusted OR (95% CI) |
|---|---|
| **ΔBT** | **1.0390 (1.0112–1.0676) *** |
| \|ΔBT\| | 1.0206 (0.9889–1.0533) |
| ΔHR | 1.0006 (0.9994–1.0019) |
| \|ΔHR\| | 0.9992 (0.9978–1.0006) |
| ΔSBP | 0.9999 (0.9992–1.0007) |
| **\|ΔSBP\|** | **1.0012 (1.0004–1.0021) *** |
| ΔDBP | 0.9992 (0.9982–1.0002) |
| \|ΔDBP\| | 1.0009 (0.9998–1.0021) |
| ΔRR | 1.0015 (0.9967–1.0063) |
| \|ΔRR\| | 0.9976 (0.9927–1.0025) |
| ΔSpO2 | 1.0007 (0.9966–1.0048) |
| \|ΔSpO2\| | 1.0016 (0.9971–1.0060) |
| ΔGCS | 1.0010 (0.9906–1.0115) |
| \|ΔGCS\| | 0.9950 (0.9838–1.0063) |
| ΔPP | 1.0006 (0.9997–1.0016) |
| **\|ΔPP\|** | **1.0014 (1.0003–1.0025) *** |
| ΔSI | 1.1001 (0.9862–1.2272) |
| \|ΔSI\| | 1.1077 (0.9758–1.2574) |

OR: odds ratio, CI: confidence interval, BT: body temperature, HR: heart rate, SBP: systolic blood pressure, DBP: diastolic blood pressure, RR: respiratory rate, SpO2: percutaneous arterial oxygen saturation, GCS: Glasgow Coma Scale, PP: pulse pressure, SI: shock index.

| | indicates the absolute value.

* Significant results based on the 95% CI values.

Bold font is used to indicate independent predictors after adjustment for age, sex, transport time, oxygen use, and the in-hospital values for BT, HR, SBP, DBP, RR, SpO2, and GCS.
